# Comparison of Analgesic Method in Laparoscopic Gastrectomy Using External Oblique Intercostal Block Versus Wound Infiltration: A Randomized Controlled Trial

**DOI:** 10.3390/jcm13144174

**Published:** 2024-07-17

**Authors:** Takanori Suzuka, Nobuhiro Tanaka, Yuma Kadoya, Takayuki Yamanaka, Mitsuru Ida, Hiroshi Nakade, Tomohiro Kunishige, Sohei Matsumoto, Naoki Ozu, Masahiko Kawaguchi

**Affiliations:** 1 Department of Anesthesiology, Nara Medical University, Kashihara 634-8522, Japan; szk.tknr.0825@naramed-u.ac.jp (T.S.); nwnh0131@naramed-u.ac.jp (M.I.); drjkawa@naramed-u.ac.jp (M.K.); 2 Department of Anesthesiology, Ikeda City Hospital, Ikeda 563-8510, Japan; kadoyayuuma@yahoo.co.jp; 3 Department of Anesthesiology, Nara Prefecture General Medical Center, Nara 630-8054, Japan; yamachan_19881120@naramed-u.ac.jp; 4 Department of Surgery, Nara Medical University, Kashihara 634-8522, Japan; nakad@naramed-u.ac.jp (H.N.); tkuni0118@naramed-u.ac.jp (T.K.); msohei@naramed-u.ac.jp (S.M.); 5 Institute for Clinical and Translational Science, Nara Medical University Hospital, Kashihara 634-8522, Japan; nao.oz@naramed-u.ac.jp

**Keywords:** nerve block, pain management, postoperative, postoperative complications, regional anesthesia

## Abstract

**Background**: External oblique intercostal block (EOIB) is effective in relieving pain in the T6 to T10 dermatomes; however, there is limited evidence comparing EOIB with conventional regional anesthesia. In this randomized controlled trial, it was hypothesized that EOIB would provide more effective analgesia than wound infiltration (WI) in laparoscopic gastrectomy. **Methods**: Thirty-two patients (aged 20–85 years) with American Society of Anesthesiologists Performance Status of 1 to 2, scheduled for laparoscopic gastrectomy, were randomly divided into EOIB and WI groups. Both procedures were performed using 40 mL of 0.25% levobupivacaine. The primary outcome was the numerical rating scale (NRS) score 12 h postoperatively. Secondary outcomes were NRS at 2, 24, and 48 h postoperatively, variation in quality of recovery-15 score on postoperative days 1 and 2 from preoperative baseline, postoperative fentanyl consumption, and variation in World Health Organization Disability Assessment Scale 2.0 scores between baseline and 3 months postoperatively. Plasma concentrations of levobupivacaine and pinprick sensation in the T4–11 dermatomes post-EOIB were evaluated to determine the exploratory endpoints. **Results**: There were no differences in the NRS scores 12 h postoperatively at rest and during movement between the EOIB and WI groups (mean standard deviation 1.3 [1.1] vs. 1.5 [1.4] and 3.1 [1.6] vs. 3.8 [1.7], respectively). Secondary outcomes did not differ significantly between the groups. The time to peak plasma concentration of levobupivacaine following EOIB was 45 min. **Conclusions**: No significant differences in NRS scores were observed between the analgesic effects of EOIB and WI at 12 h postoperatively.

## 1. Introduction

Gastric cancer was ranked as the 5th most common cancer and 4th leading cause of cancer-related deaths worldwide in 2020 [1]. However, in the absence of up-to-date protocols for laparoscopic gastrectomy for gastric cancer, there is no consensus regarding regional anesthetic techniques [2].

The external oblique intercostal block (EOIB) is a novel nerve block indicated for upper abdominal procedures that was initially reported by Elsharkawy in 2021 [3]. It blocks the anterior and lateral cutaneous branches of the T6–T10 intercostal nerves. EOIB can be performed easily under ultrasound guidance due to the superficial puncture compared to other conventional techniques, such as the rectus sheath or transverse abdominal plane blocks [3,4]. Recently, Korkusuz has demonstrated the efficacy of EOIB in laparoscopic cholecystectomy in a randomized control trial comparing EOIB to standard multimodal analgesia [5]; however, there are no reports comparing EOIB with existing regional anesthesia techniques, including wound infiltration (WI).

WI is a simple and cost-effective approach that provides adequate somatic blockade [6] and is one of the most popular regional anesthetic techniques recommended in many procedure-specific analgesic protocols [7,8,9]. However, interfascial plane block, such as transversus abdominis plane (TAP) block, has been known to improve pain relief at 12 h postoperatively compared to WI [10]. Therefore, we hypothesized that EOIB, as an interfascial plane block, may result in greater improvement in pain scores compared to WI.

We conducted a randomized controlled trial to compare EOIB and WI in laparoscopic gastrectomy. Furthermore, we examined the chronological changes in plasma local anesthetic concentration and conducted a pinprick study to investigate the analgesic efficacy of EOIB as the core outcome of peripheral regional anesthesia research [11,12].

## 2. Materials and Methods

### 2.1. Study Design and Participants

This prospective randomized controlled trial was approved by the Nara Medical University Certified Review Board (Kashihara, Nara, Japan No. 0040; approval date, 24 August 2022) and registered with the Japan Registry of Clinical Trials (jRCT) Registry on 22 September 2022 (jRCTs051220096; https://jrct.niph.go.jp/latest-detail/jRCTs051220096). The date of enrollment for the first patient was 15 November 2022. Patients (aged 20–85 years) classified according to the American Society of Anesthesiologists Performance Status 1 to 2 (ASA-PS 1–2), scheduled for robot-assisted or laparoscopic gastrectomy, and who provided written informed consent were eligible for inclusion. The following exclusion criteria were applied: emergency surgery, preoperative opioid administration, allergies to local anesthetics, inability to provide informed consent, coagulation disorders, weight <34 kg, preoperative antithrombotic therapy without adequate withdrawal duration according to the Japanese guidelines for regional anesthesia and nerve block during antithrombotic therapy, and patients deemed unsuitable to participate in the study by the investigators. The patients were randomly assigned in a 1:1 ratio to either the intervention (EOIB) or control (WI) groups. The groups were stratified by block randomization using GraphPad QuickCalcs (https://www.graphpad.com, accessed on 21 October 2022), adjustment for factors for the surgical technique (robot-assisted or laparoscopic), and type of resection site (total or pyloric gastrectomy).

### 2.2. External Oblique Intercostal Block and Wound Infiltration Procedures

(A) External oblique intercostal block group

Following anesthesia induction, EOIB was performed using a linear probe (6–13 MHz) of EDGE II (Fujifilm Sonosite, Tokyo, Japan) with the patient in a supine position. The procedures were performed by four anesthesiologists skilled in nerve blocks (TS, NT, YK, and TY). The probe was placed in the sagittal plane between the midclavicular and anterior axillary lines, at the level of the 6th rib. This was identified by counting up from the 10th rib at the level of the lower costal margin or by counting down from the 1st rib under the clavicle with ultrasound guidance. The skin entry point of the needle was between the 6th and 7th ribs, with the needle directed cephalad towards the caudal direction. The tip of the needle was placed in the EOI plane, and correct placement was confirmed by hydrodissection with a small amount of saline. Subsequently, the needle tip was advanced caudally, as in the original procedure [3], and 20 mL of 0.25% levobupivacaine per side (total 40 mL) was injected to provide local anesthesia.

(B) Wound infiltration group

The surgeon administered 40 mL of levobupivacaine (0.25%) to the peritoneum, fascia, and subcutaneous tissue via five port holes using a 23-gauge short needle before closure. There was one camera port at the umbilicus, two 5–8 mm ports on the right abdomen, and two ports on the left abdomen: one 5–8 mm and one 12 mm. An 8-mm port was used for robotic surgery [13]. In all cases, specimens are removed through a 3–5 cm longitudinal incision, including the midline umbilical port hole.

### 2.3. Intraoperative Procedure

All participants underwent general anesthesia with 1.5–2 mg/kg propofol and 0.9 mg/kg rocuronium, fentanyl, and remifentanil for induction and were maintained with sevoflurane, remifentanil, and fentanyl. Sevoflurane was administered to achieve a bispectral index of 40–60. Remifentanil was adjusted to maintain a high-frequency variability index (Mdoloris Medical Systems, Loos, France) of 50–70, which is a nociception monitor similar to the Analgesia Nociception Index; however, if a high remifentanil flow rate was needed, the use of fentanyl was considered, with the final decision made at the discretion of the attending anesthesiologist. Rocuronium was administered to maintain deep muscle relaxation during surgery using train-of-four monitoring. Remifentanil was administered at the discretion of the anesthesiologist. Fentanyl (2–4 µg/kg), acetaminophen (1 g or 15 mg/kg if the body weight was <50 kg), and ondansetron (4 mg) were administered intravenously before the end of surgery. The patient was extubated only when the train-of-four ratio was >100%, with sufficient reversal using sugammadex.

### 2.4. Postoperative Management

After termination of anesthesia, patients received intravenous fentanyl patient-controlled analgesia by CADD**^®^**-Solis PIB (Smiths Medical, St. Paul, MN, USA), with a fentanyl concentration of 0.5 µg/kg/mL, no basal flow, a bolus 1 mL on demand, and a 10-min lock-out interval. After surgery, patients received acetaminophen (every 6 h for 24 h and thereafter on demand) and nonsteroidal anti-inflammatory drugs on demand.

### 2.5. Postoperative Plasma Levobupivacaine Measurement

The collected blood samples were immediately centrifuged at 1500× *g* at 4 °C for 10 min, and the plasma samples were stored at −20 °C until further use. Plasma concentrations were measured using previously reported methods (Maruishi Pharmaceutical Corporation, Osaka, Japan) [14]. Maruishi Pharmaceutical Corporation, which produces and distributes levobupivacaine in Japan, provided non-financial support to our hospital by measuring the plasma levobupivacaine concentration in patient samples free of charge. The company had no further involvement in the study.

### 2.6. Outcomes

The primary endpoint was the numeric rating scale (NRS) score 12 h after surgery. The secondary endpoints were as follows: NRS scores at 2 h postoperatively and on postoperative days (POD) 1 and 2, postoperative fentanyl consumption, variations in quality of recovery (QoR)-15 scores from preoperative baseline to POD 1, 2, and 7, and variations in World Health Organization Disability Assessment Scale 2.0 (WHODAS 2.0) scores between baseline and 3 months post-surgery. Plasma levobupivacaine concentration was measured at multiple time points (1, 2.5, 5, 7.5, 10, 12.5, 15, 20, 30, 45, 60, 90, and 120 min) after EOIB to determine the exploratory endpoint. Pinprick sensation in the T4–11 region was assessed, with a test score of 0 denoting loss of sensation, 1 denoting decreased sensation, and 2 denoting normal sensation. A score of 0 or 1 was considered effective. Ward nurses evaluated all outcomes on POD 0, whereas the outcomes for POD 1 and 2 were assessed by a non-study-affiliated acute pain service team at our institution, who were blinded to the study [12]. The pinprick test was performed by a co-investigator who was blinded to the patients’ trial allocation.

### 2.7. Statistical Analyses

Differences in the primary endpoints between the groups were assessed using the unpaired *t*-test. The means and 95% confidence intervals (CIs) of each group and the differences between the groups were estimated. Each secondary endpoint was calculated as means with 95% CIs. Changes in the endpoints that differed between the groups were also estimated as means with 95% CIs, and *p*-values were calculated using the unpaired *t*-test for continuous values and the chi-squared test for categorical values. Plasma levobupivacaine concentrations were summarized as means with 95% CIs per time point. Peak plasma concentration (C_max_), time to peak plasma concentration (T_max_), and other parameters were determined directly from the measured values, and mean C_max_ and T_max_ values were calculated for each patient. Furthermore, variations in plasma concentrations for each participant were described using a spaghetti plot. The results of the pinprick tests were summarized using a bar graph. For the primary endpoint, a two-sided *p* < 0.05 was considered statistically significant. For the other endpoints, two-sided *p*-values were calculated, but statistical significance was not determined; hence, no multiplicity adjustment was performed.

### 2.8. Sample Size Calculation

No reference data are available for EOIB since it is a novel anesthetic method. Therefore, we assumed that the analgesic effect of EOIB compared to that of WI would be a mean improvement (reduction) of 1 on the NRS with a standard deviation (SD) of 0.9, consistent with the results of a previous study on WI in gastrectomy owing to gastric cancer [15]. The evidence for the change of 1 in the NRS as a minimal clinically important difference (MCID) was extrapolated from a report indicating that a change of 10 in the 100 mm pain visual analog scale would be the MCID for postoperative acute pain. Assuming a significance level of 5% (two-sided) and 80% power, 14 patients per group were required for comparisons using unpaired *t*-tests. Assuming a dropout rate of approximately 10%, 32 patients were randomly assigned to the two groups.

## 3. Results

### 3.1. Recruitment and Exclusions

**Figure 1:**
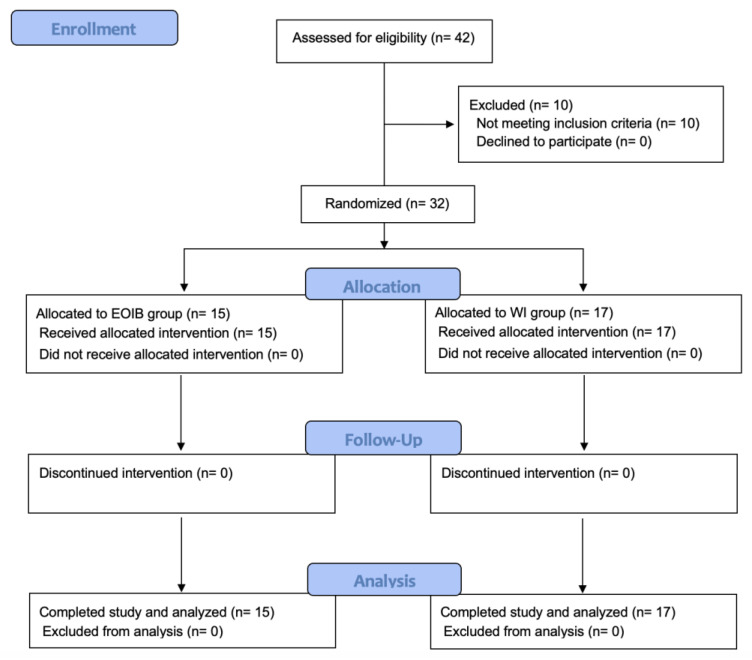
Flow diagram of patient selection. EOIB, external oblique intercostal block; WI, wound infiltration.

The CONSORT flowchart for this study is shown in Figure 1. Among the 42 patients screened between November 2022 and October 2023, 10 were excluded before randomization as they did not meet the inclusion criteria: four with ASA-PS ≥ 3, four were aged ≥85 years, one had a coagulation disorder, and one patient with deferred gastrectomy owing to the COVID-19 infection. In total, 32 patients (15 in the EOIB group and 17 in the WI group) were included in this study.

### 3.2. Patient Characteristics and Intraoperative Data

Patient characteristics and surgical duration are presented in Table 1. Data are presented as mean (SD) [16].

### 3.3. Primary Outcome

**Figure 2:**
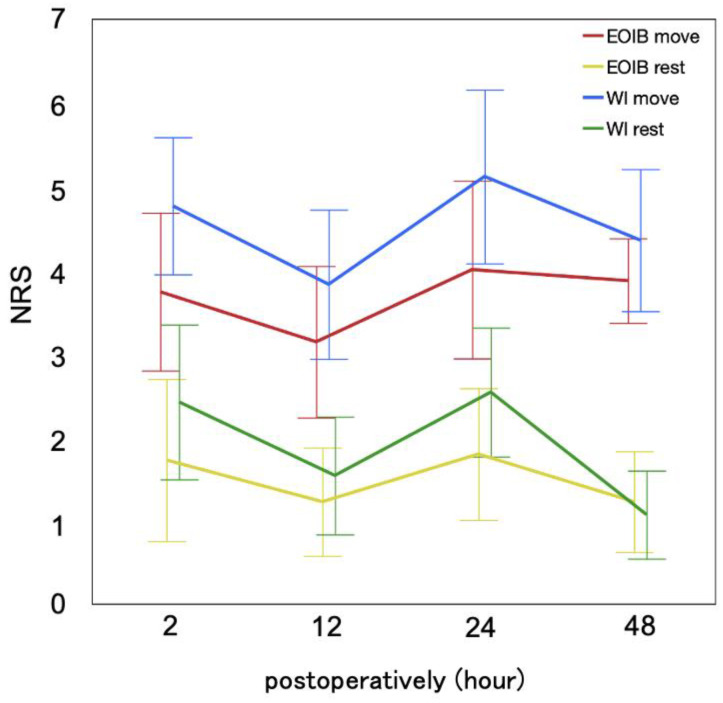
Mean NRS scores at 2, 12, 24, and 48 h postoperatively for both groups. Error bars representing 95% confidence intervals. EOIB, external oblique intercostal block; NRS, numerical rating scale; WI, wound infiltration.

There were no differences in the NRS scores 12 h postoperatively at rest and during movement between the two groups: EOIB vs. WI, mean (SD), 1.3 (1.1) vs. 1.5 (1.4), and 3.1 (1.6) vs. 3.8 (1.7), respectively (*p* > 0.05). The mean differences between the two groups (EOIB-WI) were −0.26 (95% CI: −1.17 to 0.64; *p* = 0.56) at rest and −0.69 (95% CI: −1.92 to 0.54; *p* = 0.26) during movement (Table 2 and Figure 2).

### 3.4. Secondary Outcomes

Secondary outcomes are shown in Table 2. The mean changes in the NRS scores at rest at 2, 24, and 48 h post-surgery were −0.68, −0.73, and 0.27 (*p* = 0.28, 0.16, 0.47), respectively, whereas those during movement were −1.03, −1.12, and −0.49 (*p* = 0.09, 0.12, 0.32), respectively. The mean differences in the QoR-15 scores between the two groups on POD 1, 2, and 7 from the preoperative baseline were −1.3 (95% CI: −18.9 to 16.3; *p* = 0.88), −2.4 (95% CI: −17.8 to 12.9; *p* = 0.75), and −3.1 (95% CI: −12.3 to 6.1; *p* = 0.50), respectively. There were no significant differences in any of the secondary outcomes between the two groups, including postoperative fentanyl consumption and WHODAS 2.0 scores.

### 3.5. Exploratory Outcomes

**Figure 3:**
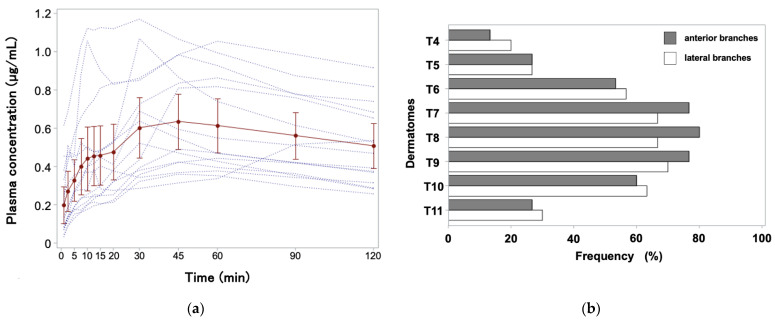
(**a**) Time courses of the plasma levobupivacaine concentrations after performing EOIB. The dashed lines indicate individual cases and the solid line indicates the average. Error bars represent 95% confidence intervals; (**b**) Frequency of dermatomes blocked on the lateral and anterior cutaneous branches of the intercostal nerves at 2 h postoperatively by EOIB. EOIB, external oblique intercostal block.

Figure 3a shows the time course of the arterial levobupivacaine concentration for 120 min after performing EOIB. The mean C_max_ and T_max_ were 0.70 μg/mL (95% CI: 0.55 to 0.86) and 44.7 min (95% CI: 30.0 to 59.3), respectively. The highest individual peak plasma concentration was 1.17 μg/mL at 30 min.

We confirmed decreased or no painful sensations in all patients postoperatively. Figure 3b illustrates the area of sensory block induced by bilateral EOIB injections. The pinprick test results suggested an effect on the lateral and anterior cutaneous branches of the intercostal nerves. Sensory blockade between T7 and T9 was observed in over 21 sides (70%), whereas that between T6 and T10 was observed in <70% of the cases. Loss of pinprick sensation involved a mean of 4.0 dermatomes (range: 0–7) in the lateral cutaneous branches and 4.1 dermatomes (range: 0–6.5) in the anterior cutaneous branches of the intercostal nerves with upper and lower limits of T4 and T11, respectively, 2 h after surgery in the EOIB group.

## 4. Discussion

In this randomized controlled trial comparing EOIB with WI as a regional anesthetic procedure during laparoscopic gastrectomy, there was no significant difference in the NRS score at 12 h postoperatively. Additionally, no significant differences were observed in the NRS scores at other time points, QoR-15 scores, or for postoperative opioid consumption.

The reason for these results could be an inadequate analgesic range of EOIB. To achieve analgesia at the umbilical port site at the T10 level, T9 and T11 must also be covered because of the presence of the TAP plexus, an abdominal neuronetwork [17]. Considering the pinprick tests performed, it is possible that T9 and T11 were not adequately covered. The target plane for local anesthetic administration in EOIB remains controversial. EOIB targets the anterior and lateral cutaneous branches of the thoracoabdominal nerves and is performed by injecting local anesthetic between the external oblique and intercostal muscles in the same interfascial plane as the thoracoabdominal nerve block through the perichondrial approach (TAPA), which is injected cephalad to the costal cartilage [18]. Ohgoshi et al. administered a cephalad injection of TAPA and found no analgesic efficacy in a pinprick test [19]. The authors debated the significance of administering the local anesthetic to the same interfascial plane as EOIB. Their results were different from those of our study but may be related to the direction of drug administration. Further investigation of these anatomical findings is required.

We must also consider the temporal disparities in administering nerve blocks. To compare pain scores more accurately, the timing of nerve block delivery in both groups must be aligned. WI is usually performed at the end of surgery [20]. However, the safety of postoperative EOIB necessitates meticulous monitoring based on information regarding local anesthetic plasma concentration levels. It was not clear whether EOIB, which involves the administration of local anesthesia in the vicinity of the intercostal muscles, would result in rapid absorption, as in an intercostal nerve block, or whether it would follow a pattern similar to that of other interfascial plane blocks such as TAP block and TAPA [21]. Furthermore, few hospitals in Japan have a post-anesthesia care unit [22], and even in our institution, patients are required to return to the general ward after surgery. Therefore, we prioritized safety, performed EOIB preoperatively, and measured the plasma concentration trends of levobupivacaine as an exploratory item.

To the best of our knowledge, this is the first study to investigate chronological changes in plasma levobupivacaine concentration following EOIB. T_max_ was 45 min in most cases; however, in one patient, T_max_ was 120 min or later (Figure 3a). This discrepancy was considered to be a result of individual differences in the diffusion of the local anesthetic solution, as variations in the ease of fascial hydrodissection have been previously demonstrated [23]. No cases exceeded the toxic level (2.62 μg/mL) [24]. Furthermore, no symptoms of local anesthetic systemic toxicity were observed in any of the patients.

Dermatomal evaluation is a core outcome of studies on regional anesthesia [11]. The results of the pinprick test suggested that a sensory block area was present in both the lateral and anterior branches of the thoracoabdominal nerves (Figure 3b), consistent with previous studies [3]. Variation among cases has been attributed to individual differences in the interfascial plane block [23]. Although we followed the method described by Elsharkawy, the uniformity of the procedure in the novel block may be incomplete, and further investigation is required [21,25,26].

This study had some limitations. First, it was conducted at a single institution and focused only on laparoscopic gastrectomy. Second, the targeted surgical procedures were varied. At our institution, we excluded proximal gastrectomy because it is more invasive owing to the intraoperative insertion of esophagogastroduodenoscopy. Owing to the limited number of cases, we included total gastrectomy. Considering these factors, block randomization was used, resulting in two cases of total gastrectomy in each group.

Third, blinding of the anesthesiologist was not possible because of the different timings of performing the block. The scheduling of each procedure was consistent with that in previous studies and was performed at the appropriate time for each surgery, with safety as a priority. Fourth, compared to previous reports, we used a smaller volume of local anesthetics for EOIB [5]. In this study, it was necessary to use the same amount of local anesthetic as in WI in order to equalize the conditions for the systemic absorption of local anesthetics. Therefore, this result may underestimate the effectiveness of EOIB. Further research to determine the optimal volume for EOIB is warranted.

## 5. Conclusions

No significant differences were observed between WI and EOIB in laparoscopic gastrectomy for gastric cancer. Further studies are needed to clarify the analgesic effects and mechanisms of action of EOIB.

## Figures and Tables

**Table 1:** Patient characteristics and intraoperative data.

	EOIB Group (*n* = 15)	WI Group (*n* = 17)
Patient characteristics		
Age (years)	68.2 (10.5)	70.6 (8.3)
Sex, *n* (%)		
Male	11 (73.3)	14 (82.3)
Female	4 (26.7)	3 (17.6)
Weight, kg	63.8 (11.0)	55.7 (10.3)
Height, cm	162.5 (9.6)	161.4 (6.0)
BMI, kg/m^2^	24.1 (3.0)	21.4 (4.0)
ASA-PS, *n* (%)		
I	0 (0)	2 (12)
II	15 (100)	15 (88)
Albumin (g/dL)	4.2 (0.5)	3.9 (0.6)
Surgical data		
Type of surgery, *n* (%)		
Laparoscopy	11 (73.3)	13 (76.5)
Robot-assisted Laparoscopy	4 (26.7)	4 (23.5)
Type of gastrectomy, *n* (%)		
Total	2 (13.3)	2 (11.8)
Distal	13 (86.7)	15 (88.2)
Duration of surgery, min	324.9 (84.8)	316.4 (81.9)
Duration of anesthesia, min	398.3 (87.6)	378.2 (88.6)
Intraoperative fentanyl use, µg	436.7 (127.4)	394.1 (76.8)
Remifentanil total amount (µg/kg/min)	0.17 (0.06)	0.16 (0.05)

Data are presented as mean (SD) or number (percent). ASA-PS, American Society of Anesthesiologists physical status; BMI, body mass index; EOIB, external oblique intercostal block; SD, standard deviation; WI, wound infiltration.

**Table 2:** Outcomes.

	EOIB Group (*n* = 15)	WI Group (*n* = 17)	Mean Difference (EOIB—WI) (95% CI)	*p*-Value
Primary Endpoint				
NRS scores				
12 h at rest	1.27 (1.10)	1.53 (1.37)	−0.26 (−1.17 to 0.64)	0.56
12 h with movement	3.13 (1.64)	3.82 (1.74)	−0.69 (−1.92 to 0.54)	0.26
Secondary Endpoint				
NRS scores at rest				
2 h	1.73 (1.62)	2.41 (1.80)	−0.68 (−1.92 to 0.57)	0.28
24 h	1.80 (1.32)	2.53 (1.50)	−0.73 (−1.76 to 0.30)	0.16
48 h	1.33 (1.11)	1.06 (1.03)	0.27 (−0.50 to 1.05)	0.47
NRS scores with movement				
2 h	3.73 (1.71)	4.76 (1.60)	−1.03 (−2.23 to 0.16)	0.09
24 h	4.00 (1.93)	5.12 (2.03)	−1.11 (−2.55 to 0.32)	0.12
48 h	3.87 (0.92)	4.35 (1.66)	−0.49 (−1.47 to 0.50)	0.32
QoR-15				
Pre	141.53 (10.79)	137.94 (16.70)	-	-
POD 1—Pre	−24.2 (25.2)	−22.9 (23.6)	−1.32 (−18.94 to 16.31)	0.88
POD 2—Pre	−16.6 (19.9)	−14.2 (22.3)	−2.42 (−17.75 to 12.91)	0.75
POD 7—Pre	−4.5 (14.8)	−1.4 (10.6)	−3.11 (−12.33 to 6.10)	0.50
Postoperative fentanyl				
Total amount (µg/kg)	10.12 (10.54)	9.88 (6.86)	0.24 (−6.10 to 6.59)	0.94
WHO-DAS 2.0 (%)				
Pre	4.72 (4.42)	10.66 (16.20)	-	-
3 months	11.25 (17.52)	16.79 (19.28)	0.40 (−8.20 to 9.00)	0.92
Exploratory Endpoint				
Plasma concentration of levobupivacaine				
C_max_ (µg/mL)	0.70 (0.55 to 0.86)	-	-	-
T_max_ (min)	44.7 (30.0 to 59.3)	-	-	-

Values in each group are expressed as mean (SD) or number (percent). C_max_ and T_max_ are expressed as the mean with a 95% CI. CI, confidence interval; SD, standard deviation; C_max_, peak plasma concentration; EOIB, external oblique intercostal block; NRS, numerical rating scale; QoR-15, quality of recovery-15; POD, postoperative day; WHO-DAS, World Health Organization Disability Assessment Schedule; Pre, preoperative day; T_max_, time to peak plasma concentration; WI, wound infiltration.

## Data Availability

The data that support the findings of this study are available from the corresponding author, upon reasonable request.
